# The association between medical student research engagement with learning outcomes

**DOI:** 10.1080/10872981.2022.2100039

**Published:** 2022-07-11

**Authors:** Guoyang Zhang, Hongbin Wu, A’Na Xie, Huaqin Cheng

**Affiliations:** a School of Health Professions Education, Faculty of Health, Medicine and Life Sciences, Maastricht University, Maastricht, the Netherlands; b National Centre for Health Professions Education Development, Peking University, Beijing, China; c Institute of Medical Education, Peking University, Beijing, China; d The Fourth Affiliated Hospital, The Fourth Affiliated Hospital, Zhejiang, China

**Keywords:** Medical student, research engagement, learning outcomes, China

## Abstract

Medical student research engagement has been considered as a critical component of undergraduate medical education. However, evidence on the association between medical student research engagement with learning outcomes is lacking. The objectives of our study are: (1) to outline the landscape of medical student research engagement in China; (2) to explore the association between medical student research engagement and learning outcomes, and whether this association is different among students with different characteristics. A paper questionnaire was developed, piloted, and administered to medical students at 33 medical schools in China. Research engagement was measured by the times students engaged in research projects while learning outcomes referred to learning outcomes contained in the Standards for Basic Medical Education in China. Chi-square tests were used to measure statistical significance between research engagement and the characteristics of participants. We analysed relationships between research engagement and learning outcomes using multivariate linear regression with medical school fixed effects. The overall response rate was 86.7%. 10,062 medical students completed the questionnaire, 55.5% of which had participated in one or more research projects. Research engagement differed by the length of the program, gender, and academic performance. Research engagement was also positively associated with students’ overall learning outcomes, especially in the Science and Scholarship domain (once, β = 0.20, *P <* 0.001; twice or more, β = 0.43, *P* < 0.001) and the Professionalism domain (once, β = 0.12, *P* < 0.05; twice or more, β = 0.25, *P* < 0.01). The relationships between research engagement and learning outcomes differed significantly by gender. Medical student research engagement is significantly positively associated with medical students’ learning outcomes, especially in the Science and Scholarship domain and the Professionalism domain. Besides, men benefit more from engaging in research projects, particularly in the Science and scholarship domain.

## Introduction

Student engagement has been received increased attention in medical education globally during the past decades [1–3]. The term is generally accepted as ‘the interaction between the time, effort and other relevant resources invested by both students and their institutions intended to optimize the student experience and enhance the learning outcomes and development of students and the performance, and reputation of the institution.’[4]. The ASPIRE to Excellence Initiative developed by Association for Medical Education in Europe divides medical student engagement into four domains: engagement in policy and decision-making activities at the school, engagement in the provision and evaluation of the school’s education program, engagement in the academic community, and engagement in the local community, extracurricular activities, or service delivery [5]. Medical student research engagement (MSRE), referring to students participating in research projects or attending academic conferences, has been considered as a key domain of student engagement [5].

MSRE enables students to develop skills related to evaluating information critically, communicating research findings, and advancing medical knowledge, which are core learning outcomes listed by some global standards for basic medical education[6–10]. To enhance medical students’ research abilities, medical schools have provided opportunities for students to engage in research through electives, or mandatory courses, summer research programs [11]. For example, in the USA, Duke University School of Medicine and Stanford University School of Medicine have integrated research into the curriculum for more than forty years to create future physician leaders [12]. The 2020 Graduate Questionnaire conducted by the Association of American Medical Colleges indicates 83.9% of American medical graduates engaged in a research project guided by a faculty member [13]. Students selected components, aiming to develop students’ skills related to research and critical appraisal, are a requirement of undergraduate training in the UK [14]. Brazilian medical schools also have made efforts to ensure the scientific training programs available for undergraduates, and 47% of students have taken part in scientific training programs on their campus [15].

Previous studies have already investigated MSRE in some countries and have indicated that these medical research programs are positively related to student learning outcomes. Participating in research projects allows medical students to improve research-specific and interpersonal skills. Research skills include looking for literature, formulating research questions, learning new techniques, writing academic papers, and thinking critically while interpersonal skills refer to teamwork skills, communication skills, and presentation skills [16–20]. Moreover, Houlden and colleagues use a student survey to evaluate a mandatory minimum 8-week ‘critical inquiry’ program of Queen’s University School of Medicine, finding students gain an increased sense of confidence in the above abilities and the percentage of students who intend to pursue a research career grow 10%[21].

However, on the one hand, even though previous studies examine the learning outcomes medical students obtained from MSRE, these studies primarily focus on research skills that students receive, but how meaningful MRSE is to students’ other non-research skills, especially the learning outcomes relevant to graduation requirements, is still unclear [22]. On the other hand, research in MSRE, especially empirical studies of medical students in China, remains scarce compared with the studies conducted in the Western context [6,16,22]. China proposes its Accreditation Standards for Basic Medical Education in 2016, and the graduation requirements of medical students’ learning outcomes are divided into four domains (science and scholarship, clinical practice, health and society, professionalism). Under this context, we conduct this study to outline the landscape of MSRE in China, examine the relationship between MSRE and learning outcomes in the accreditation standards, and identify whether there are differences in learning outcomes obtained from MSRE between groups with different characteristics. We hope to generate fresh insight into student research engagement in Chinese context and provide suggestions for improving medical student research experience.

## Methods

### Instrument

To provide an overview of undergraduate medical education in China, the China Medical Student Survey (CMSS), a nationwide survey of medical undergraduates’ learning experiences and outcomes, was administered by National Centre for Health Professions Education Development (NCHPED, Link: https://medudata.bjmu.edu.cn/) since 2019. The whole questionnaire drew lessons from Medical School Graduate Questionnaire and National Survey of Student Engagement, both surveys administered annually in the USA and Canada [13,23].The CMSS was designed and established by experts from medicine, education, as well as sociology. It was divided into six sections (demographics, pre-college experiences, learning experiences, teaching evaluation, clinical practice, learning outcomes and employment). A pilot survey at Peking University was conducted to examine whether the whole questionnaire was qualified to be the research instrument. For our research purposes, we selected 44 items from the whole questions groupped into three domains.

The first domain was demographic and academic information. The location of students’ homes was classified as either rural or urban. Parental occupational statuses were estimated using the International Socio-Economic Index of Occupational Status (ISEI) and ranged from 16 to 88 (lowest to highest prestige) [24]. Parental educational attainments were measured as years of education. The students’ National College Entrance Examination (CEE) score was standard score from 0 to 750. Academic performance was divided into four groups (top 25%, 26–50%, 51–75% and bottom 25%). The length of education programs included eight-year programs, seven-year programs, and five-year programs. Specifically, before 2015, there were three types of educational programs in China’s medical education system according to the duration of studies, including an eight-year program in clinical medicine leading to a Doctor of Medicine degree, a seven-year program in clinical medicine leading to a Master’s of Medicine degree and a five-year program in clinical medicine leading to a Bachelor’s of Medicine degree [25]. To improve medical education quality in China, all seven-year degree programs were changed into ‘5 + 3’ master degree programs in 2015 [26]. The year 2019 witnessed that the last students attending seven-year degree program in 2014 graduated. Although different programs they were, the undergraduate medical education period in these three types of programs all lasted for five years.

The second domain, research engagement, referred to how many times medical students engaged in research projects (never; once; twice or more) during their undergraduate medical education. The third domain, learning outcomes, involved 33 outcomes based on the Accreditation Standards for Basic Medical Education in China which proposed according to the WFME Global Standards for Quality Improvement [8], WHO Guidelines for Quality Assurance of Basic Medical Education in the Western Pacific Region [7], and Global Minimum Essential Requirements in Medical Education [10]. In our study, these learning outcomes were measured by added value which meant that we compared students’ pre-college level of learning outcomes with the after-college (graduation) level. Four domains of learning outcomes were identified, and each domain was measured by 6 to 10 items using a 10-point scale. For instance, the S&S domain was measured by eight items, and its score was the average of the answers. The overall score of learning outcomes was measured by the sum of the four domains. The learning outcomes scale had a good reliability, with Cronbach’s alpha value of these four dimensions 0.88, 0.99, 0.95, 0.93 respectively. The framework of learning outcomes was given below (Table 1). Detailed items of the learning outcomes can be found in Appendix 1.

**Table 1.** The framework of learning outcomes.

Learning Outcomes (LO)	Descriptions
Science and scholarship (S&S)	Eight learning outcomes that the medical graduate as a scientist and a scholar has to obtain. (e.g., medical knowledge, academic writing skills)
Clinical practice (CP)	Ten learning outcomes that the medical graduate as a practitioner has to obtain. (e.g., physical examination skill, writing medical records)
Health and society (H&S)	Six learning outcomes that the medical graduate as a health advocate has to obtain. (e.g., awareness of advancing public health)
Professionalism (PF)	Ten learning outcomes the medical graduate as a professional has to obtain. (e.g., teamwork skills, respect for others)

### Setting and samples

This study was organized by the NCHPED and was conducted in 33 medical schools located in China. The NCHPED is commissioned by the Ministry of Education and the National Health Commission of the People’s Republic of China, has an enduring commitment to the continuous improvement of medical education in China through creating accreditation standards, accrediting degree programs in the medical disciplines, and conducting health professions education research. Undergraduate medical students who graduated in June 2019 participated the CMSS. Specifically, the sampling process had three stages. First, 20 medical institutions that were members of the Association for Health Professions Education Research in China (AHPERC), participated in the survey. Second, to be sure that the sample was representative, and that each subgroup division (e.g., the location of the institution, the reputation of the institution) was similar to the total population at medical institutions in China, the study used a purposive sampling technique to select another 13 medical schools. Finally, the probability-proportional-to-size sampling was used for medical graduates from the selected 33 institutions. It should be noted that only a small number of prestigious medical institutions had chances to provide long-term programs, so the percentage of students from seven- or eight-year degree programs was relatively small. Informed consent was obtained from all participants involved in this questionnaire. Participants were informed in advance about the use of their data for this study, and their answers were kept confidential and anonymised. In order to ensure the quality of answering, each medical school assigned research assistants to organize students to fill out questionnaires in classrooms offline. The research team collected data from 15 June 2019, to 15 July 2019. Incomplete responses were excluded.

### Ethical approval

Peking University Institutional Review Board (PKU IRB) usually exempt educational research from requirement of ethical approval. This study was granted an exemption from requiring ethics approval by PKU IRB. All methods were performed in accordance with the relevant guidelines and regulations.

### Data analysis

The data analysis began with descriptive statistics. Dichotomous variables were reported as a number (percentage), whereas continuous variables were reported as mean ± standard deviation (SD) or median (interquartile range). Next, Chi-square tests were performed to measure statistical significance between research engagement and the characteristics of participants. Finally, the multivariate linear regression models were used to explore relationships between MSRE (as the independent variable) and learning outcomes (as dependent variables). Student characteristics, treated as control variables, were controlled for by entering them into the regression models. These variables included gender, home location, family factors, CEE score, length of the education program and academic performance. To control for the differences between institutions and the missing variables at the institution level, we used medical schools as fixed effects for all models. The statistical analysis was performed using Stata MP Version 14.0 software (Stata Inc., Chicago, IL, USA). Only a two-paired p-value < 0.05 was considered to have statistical significance.

## Results

### Demographics and academic characteristics

In total, 11,596 students were approached to participate, of whom 10,062 completed the questionnaire, yielding an overall response rate of 86.7%.

Table 2 presented the summary statistics for medical students’ demographics and academic characteristics. Of the 10,062 students, 55.1% were female and 44.9% were male. Almost two-thirds (64.5%) were from urban areas, versus just over one-third (35.5%) from the rural areas. The students’ fathers and mothers had a mean occupational status index of 38.2 (SD = 20.4) and 36.2 (SD = 17.9), respectively. The mean for fathers’ education attainment was 11.5 (SD = 4.1), and that for mothers was 10.5 (SD = 4.3). Concerning academic variables, the mean and SD of the CEE score was 588.4 ± 57.6. Five-year programs had the highest proportion of students at 74.9%, compared with 15.7% in seven-year programs and 9.4% in eight-year programs. The proportion of students whose academic performance was in the top 25%, 26–50%, 51–75% and bottom 25% was 34.9%, 31.5%, 23.1% and 10.5%, respectively. Overall, the sample could be regarded nationally representative. The overall mean of learning outcomes was 15.9 ± 6.3. The means of the learning outcomes in the S&S, CP, H&S, and PF domain were 2.8 ± 1.8, 5.9 ± 2.1, 4.2 ± 2.1, and 2.8 ± 1.9, respectively.

**Table 2.** Demographics and academic characteristics of 10,062 students from 33 medical schools compared with national medical student demographics.

Characteristics	Sample (N&%, or M± SD)	Nationally(%)^[27,28]^
Gender		
Male	4518(44.9)	45.0
Female	5544(55.1)	55.0
Home location		
Urban	6487(64.5)	63.1
Rural	3575(35.5)	36.9
Family Factors		
ISEI of the father	38.19(20.4)	
ISEI of the mother	36.2(17.9)	
Education attainment of the father	11.46(4.1)	
Education attainment of the mother	10.45(4.3)	
CEE Score	588.4(57.6)	
Length of education program		
Five-year program	7532(74.9)	89.0
Seven-year program	1583(15.7)	5.6
Eight-year program	947(9.4)	1.8
Academic performance		
Top 25%	3260(34.9)	
26–50%	2493(31.5)	
51–75%	2160(23.1)	
Bottom 25%	984(10.5)	
Learning outcomes	15.9 (6.3)	
Science and scholarship (S&S)	2.8(1.8)	
Clinical practice (CP)	5.9(2.1)	
Health and society (H&S)	4.2(2.1)	
Professionalism (PF)	2.8(1.9)	

Note: Percentages may not add to 100 because of rounding. The national data was from the 2020 CMSS which involved 180,348 undergraduate medical students from 119 medical schools. All seven-year degree programs were changed into ‘5 + 3’ master’s degree programs in 2015 so national data about seven-year degree program in 2019 was corresponding to ‘5 + 3’ master’s degree programs in 2020.

### The landscape of MSRE in China

Concerning MSRE, 44.5% of those surveyed had never engaged in any research activities, 41.8% had done one research project, and 13.7% had engaged in two or more research projects during undergraduate studies. As shown in Table 3, the difference between male and female groups was significant (*P* < 0.01). The proportion of male students who were never involved in research was 44.2%, slightly lower than the proportion of female students (44.8%). The similar result was observed for participating in research programs exactly one time (41.3% vs. 43.1%); however, fewer female students than male students (12.1% vs. 15.7%) engaged in research projects two or more times. Students from different degree programs were found to have a statistically significant difference in MRSE (*P* < 0.01). Slightly more than one-tenth of graduates from the eight-year program had never been involved in research activities, while the figure for seven-year programs was around 30% and for five-year programs was more than half. Students who attended eight-year programs were more likely to take part in research activities than those in seven-year and five-year programs (88.1% vs. 68.9% vs. 48.6%). There was a significant difference among the four groups of academic performance (*P* < 0.01). The top 25% of respondents’ research engagement was 19.0% higher in comparison with the bottom 25%.

**Table 3.** The landscape of MSRE in China.

	Student engagement in the research projects			
Variables	Never (N&%)	Once (N&%)	Twice or more (N&%)	Chi-square
Research engagement	4477(44.5)	4203(41.8)	1382(13.7)	
Gender				
Male	1996 (44.2)	1813 (40.1)	709 (15.7)	28.4***
Female	2481 (44.8)	2390 (43.1)	673 (12.1)	
Length of education programs				
Five-year program	3873 (51.4)	2868 (38.1)	791 (10.5)	899.6***
Seven-year program	492 (31.1)	849 (53.6)	242 (15.3)	
Eight-year program	112 (11.9)	486 (51.2)	349 (36.9)	
Academic performance				
Top 25%	1182 (36.3)	1441 (44.2)	637 (19.5)	228.5***
26–50%	1322 (44.9)	1258 (42.8)	363 (12.3)	
51–75%	1060 (49.0)	898 (41.6)	202 (9.4)	
Bottom 25%	544 (55.3)	350 (35.5)	90 (9.2)	

Note: Chi-square tests, *** *P* < 0.01; Results for other non-significant variables are not reported.

### The relationship between MRSE and learning outcomes

Results of the multivariate linear regression (Table 4) indicated MSRE was significantly positively related to medical students’ overall learning outcomes (once, β = 0.43, *P* < 0.05; twice or more, β = 0.77, *P* < 0.01). Compared with students who never participated in research activities, those who were engaged in research projects achieved better learning outcomes in the dimensions of S&S (once, β = 0.20, *P* < 0.001; twice or more, β = 0.43, *P* < 0.001) and PF (once, β = 0.12, *P* < 0.05; twice or more, β = 0.25, *P* < 0.01). Nevertheless, no significant association was identified in the CP and H&S dimensions.

**Table 4.** The relationship between MSRE and learning outcomes (β, 95%CI).

	Overall LO	S&S	CP	H&S	PF
Research engagement					
once	0.43*	0.20***	0.04	0.08	0.12*
	(0.10, 0.76)	(0.11, 0.29)	(−0.11, 0.18)	(−0.03, 0.18)	(0.02, 0.21)
twice or more	0.77**	0.43***	0.03	0.06	0.25**
	(0.22, 1.31)	(0.27, 0.59)	(−0.18, 0.23)	(−0.11, 0.24)	(0.11, 0.39)
Female	−0.53***	0.02	−0.06	−0.17***	−0.32***
	(−0.81, −0.246)	(−0.08, 0.12)	(−0.18, 0.06)	(−0.25, −0.09)	(−0.39, −0.25)
Rural	0.06	−0.005	−0.002	0.03	0.04
	(−0.27, 0.40)	(−0.11, 0.10)	(−0.13, 0.12)	(−0.0697, 0.12)	(−0.06, 0.15)
Education attainment of the father	0.02	−0.01	0.03	0.004	−0.008
	(−0.03, 0.06)	(−0.02, 0.006)	(0.01, 0.04)	(−0.01, 0.02)	(−0.02, 0.01)
Education attainment of the mother	0.04	0.01	0.01	0.002	0.01
	(−0.01, 0.08)	(−0.002, 0.03)	(−0.003, 0.02)	(−0.01, 0.02)	(−0.004, 0.03)
ISEI of the father	−0.002	0.001	−0.004	−0.0002	0.001
	(−0.01, 0.01)	(−0.001, 0.004)	(−0.007, 0.00)	(−0.003, 0.003)	(−0.002, 0.003)
ISEI of the mother	−0.006	−0.0004	−0.002	−0.003	−0.001
	(−0.02, 0.01)	(−0.003, 0.003)	(−0.01, 0.002)	(−0.01, 0.001)	(−0.005, 0.004)
CEE Score	0.04	−0.002	0.08	0.01	−0.06
	(−0.16, 0.23)	(−0.05, 0.05)	(−0.02, 0.18)	(−0.06, 0.08)	(−0.10, −0.01)
Academic performance					
26–50%	−0.04	−0.03	−0.004	−0.04	0.03
	(−0.35, 0.27)	(−0.12, 0.06)	(−0.11, 0.10)	(−0.14, 0.06)	(−0.07, 0.14)
51–75%	−0.23	−0.10	−0.02	−0.02	−0.08
	(−0.61, 0.15)	(−0.22, 0.01)	(−0.16, 0.120)	(−0.16, 0.11)	(−0.16, 0.001)
Bottom 25%	−0.40	−0.28**	−0.01	−0.03	−0.08
	(−0.98, 0.17)	(−0.46, −0.10)	(−0.19, 0.17)	(−0.20, 0.14)	(−0.25, 0.08)
Observations	10,062	10,062	10,062	10,062	10,062

Note: Medical school fixed effects, and robust standard errors are clustered at the medical school level. *** *P* < 0.001, ** *P* < 0.01, * *P* < 0.05. Abbreviations: CI = Confidence Interval, LO = Learning Outcome, S&S = Science and Scholarship, CP = Clinical Practice, H&S = Health and Society, PF = Professionalism.

We further examined whether the association between MSRE with learning outcomes in two gender groups (Table 5). Compared with male students who was never involved in research, male students who did two or more research projects had significantly better learning outcomes (β = 0.94, *P* < 0.05), whereas such a significant result was not found among female students. In addition, participating in research programs allowed male students to significantly sharpen their knowledge of S&S (once, β = 0.28, *P* < 0.001; twice or more, β = 0.56, *P* < 0.001), while this activity only significantly increased female students’ S&S knowledge when they engaged in two or more research projects (β = 0.30, *P* < 0.01). In contrast, female students significantly increased their PF if they were engaged in research (once, β = 0.15, *P* < 0.05; twice or more, β = 0.20, *P* < 0.05); however, the male students significantly increased their PF only when the frequency of research engagement was twice or more (β = 0.28, *P* < 0.01).

**Table 5.** The relationship between MSRE and learning outcomes: by gender (β, 95%CI).

	Overall LO	S&S	CP	H&S	PF
**Male: Research engagement(N = 4518)**					
once	0.48	0.28***	0.04	0.08	0.08
	(−0.02, 0.98)	(0.16, 0.40)	(−0.18, 0.26)	(−0.10, 0.26)	(−0.08, 0.23)
twice or more	0.94*	0.56***	−0.001	0.10	0.28**
	(0.17, 1.71)	(0.34, 0.78)	(−0.33, 0.32)	(−0.13, 0.33)	(0.10, 0.46)
**Female: Research engagement(N = 5544)**					
once	0.40	0.14	0.04	0.07	0.15*
	(−0.12, 0.92)	(−0.02, 0.30)	(−0.14, 0.21)	(−0.08, 0.23)	(0.01, 0.28)
twice or more	0.56	0.30**	0.05	0.01	0.20*
	(−0.10, 1.22)	(0.12, 0.48)	(−0.18, 0.28)	(−0.21, 0.22)	(0.004, 0.40)

Note: Medical school fixed effects, and robust standard errors are clustered at the medical school level. *** *P* < 0.001, ** *P* < 0.01, * *P* < 0.05. Abbreviations: CI = Confidence Interval, LO = Learning Outcome, S&S = Science and Scholarship, CP = Clinical Practice, H&S = Health and Society, PF = Professionalism.

## Discussion

There has been a longstanding tradition of medical students’ engagement in research activities, and it has been a critical component of medical education for many years [29]. To the best of our knowledge, this study involving 10,062 participants from 33 medical schools was the first nationally representative survey of medical student research engagement in China and the first study used national data to explore the association between MRSE and learning outcomes. We mapped the landscape of MRSE in China and explore its relationship with students’ learning outcomes. Overall, over half of medical students in China had done at least one research project at college, and 44.9% were not engaged in any research activities at all. Besides, significant difference was found in research engagement between male and female students, and medical students with better academic performance and who were enrolled in long-term programs were more likely to engage in research. In addition, doing research projects was positively associated with students’ overall learning outcomes, especially in the S&S and PF domains. Further analysis also demonstrated that learning outcomes achieved by students might differ depending on gender.

Regarding Chinese students’ engagement in research at least once, the percentage is 55.1%, much lower than the USA (83.9%) [13] but 10% higher than Brazil (47%) [15]. Of note, American medical schools employ a ‘premed’ system, thus medical undergraduates hold a previous degree. Students who have research experience during this time may obtain research knowledge and skills, thus tending to engage more in research after entering medical schools [11]. This can, in part explain why the percentage of America is so higher than China. Funston et al. (2015) conducted an international survey about student research engagement at an undergraduate level and find the majority of participants from UK, New Zealand, Canada, Malaysia, and France involves in different kinds of research activity [30]. Clearly, no matter in developed countries or developing countries, MSRE has been a key part of medical education around the world.

Besides, China medical students’ research engagement also varies with different programs. Students in eight- and seven-year programs are more likely to participate in research activities during their undergraduate medical education than those in five-year programs. This discrepancy can be attributed to the influence of medical institutions. As previously stated, only leading medical institutions in China have opportunities to provide eight-year or seven-year programs. Such leading universities are usually research-oriented, attaching more importance to research training and providing students with greater research funding. This result agrees with Li’s findings that Chinese students from elite colleges have higher levels of research participation [31].

Furthermore, this study finds research experience fosters students’ learning outcomes, particularly in the S&S and PF domains. The results are in line with those of previous studies suggesting students involve in research tend to gain more medical knowledge, research-related skills, and interpersonal skills [6,16,19,22]. MSRE is not significant related with the CP and H&S domains, however. A possible explanation for this may be that the projects which students do are associated with basic science and social science, but not related to clinical issues. Therefore, less knowledge and skills of CP and H&S domains are learned through research activities. Since clinical research are also important for all health professionals, medical schools may need act to offer more clinical research projects to medical undergraduates.

Regarding gender disparity, significant difference exists in research participation between male and female students. This finding is consistent with previous studies conducting at Saudi Ariba which suggests that participation in research significantly differed by gender [32], but contrary to that of Park et al (2010) do at New Zealand [33]. More evidence is needed in the future. Interestingly, male students benefit more concerning the S&S domain of learning outcomes. A study conducted at Ireland medical schools shows undergraduate male medical students report feeling significantly more competent with regard to research-related skills (e.g., study design, biological statistics, paper presenting) and transferable skills (e.g., communication skills, teamwork skills, problem-solving skills), which is consistent with our study findings [11]. One possible explanation is male medical students are more likely to involve more in research [30]. More research engagement may lead to more learning outcomes obtained from involvement in research. Reasons behind this phenomenon need further investigations in further studies.

There are some limitations to our study. First, the cross-sectional study has some weaknesses because it produces less evidence than a longitudinal design. Second, this study is limited by its use of self-assessment tools, as students may overestimate or underestimate their learning outcomes. Nevertheless, due to the large sample size of this investigation, this issue might have a limited impact on the accuracy of the results. Finally, as with other questionnaire studies, the use of a questionnaire rather than a qualitative research method increased the scope of the study but limited its depth. The reasons why males and females are engaged differently in research activities and the reasons why male students gain more learning outcomes from research involvement needs further qualitative studies. Notwithstanding these limitations, the study adds to our understanding of medical students’ research engagement in China and its relationship to learning outcomes. To develop a full picture of MRSE, it may be possible to employ qualitative research method to explore the process of MRSE more depth, outlining the mechanism between MRSE and learning outcomes in future. Additional research should also be undertaken to design an instrument about the learning outcomes from MRSE so that medical schools are able to measure the effectiveness of their research programs.

## Conclusion

This study has shown that over half of the medical students in China are engaged in research projects. MSRE is related to the length of programs and academic performance. Research engagement is positively associated with medical students’ overall learning outcomes, especially in the S&S and PF dimensions. Our findings also suggest that significant difference exists in research participation between male and female students, and men benefit more from engaging in research projects, particularly in the Science and Scholarship domain.

## Data Availability

The datasets used and/or analyzed during the current study are available from the corresponding author on reasonable request.

## Appendix 1: Selected Items from China Medical Student Survey

**Demographic and Academic Information** (8 items)

1. What is your gender identity?

- Man
- Woman
  2. What is your home location?

- Urban
- Rural

3. What is your father’s career?

- Government administrator
- Enterprise senior management
- Professional (such as teacher/doctor)
- Technical Support Staff (such as Technician/Nurse)
- General Management and Clerical Staff
- Business, Service Personnel
- Self-employed households
- Private Entrepreneur
- I. Farmer (Forestry/ Husbandry/Fishing)
- Worker (Production and Transportation Equipment Operators)
- Migrant Workers
- Retired
- The unemployed
- Other (Please Specify)
  4. What is your mother’s career?

- Government administrator
- Enterprise senior management
- Professional (such as teacher/doctor)
- Technical Support Staff (such as Technician/Nurse)
- General Management and Clerical Staff
- Business, Service Personnel
- Self-employed households
- Private Entrepreneur
- I. Farmer (Forestry/ Husbandry/Fishing)
- Worker (Production and Transportation Equipment Operators)
- Migrant Workers
- Retired
- The unemployed
- Other (Please Specify)

5. What is the level of education completed by your father?

- Below Elementary school
- Junior High School
- High School or secondary vocational education
- Higher vocational and undergraduate education
- Bachelor or above

6. What is the level of education completed by your mother?

- Below Elementary school
- Junior High School
- High School or secondary vocational education
- Higher vocational and undergraduate education
- Bachelor or above

7. Please write your National College Entrance Examination (CEE) score______.
8. What is the length of your education program?
  - Five-year program
  - Seven-year program
  - Eight-year program
9. What is your overall GPA?

- Top 25%
- 26–50%
- 51–75%
- Bottom 25%

10. What is your medical school location?

- Municipalities
- Eastern
- Central
- Western

**Research engagement** (1 items)

11. How many times are you engaged in academic research projects?

- Never
- Once
- Twice or more

**Detailed items of the Learning Outcomes** (33 items)

12. What is your actual level in terms of the following learning outcomes? Please write the pre-college and after-college level.

Response options: 1 = Poor to 10 = Excellent, 0 = I don’t have it.

**Table:**

Overall learning outcomes	Pre-college	After-college
**Science and scholarship (S&S)**		
1. The fundamental knowledge and methods of natural sciences such as mathematical and chemical disciplines		
2. The fundamental knowledge and methods of humanities and social sciences		
3. The fundamental knowledge and methods of medicine		
4. Understand the etiology, clinical features, diagnosis, treatment and prognosis of common presentations at the stages of life		
5. Mastery and application the knowledge of traditional Chinese medicine		
6. Academic writing ability		
7. Mathematical statistical analysis ability		
8. Critical thinking		
**Clinical practice (CP)**		
1. Take a medical history		
2. Perform a physical examination		
3. Write medical records		
4. Perform disease diagnosis and differential diagnosis based on medical history, physical examination, auxiliary examination		
5. Be able to select appropriate clinical examination methods according to the actual situations of patients		
6. Be able to help determine patients’ treatment plans and explain the rationality		
7. Ability to integrate knowledge such as disease prevention and health maintenance into clinical practices		
8. Assess the extent and changes of patients’ condition		
9. First aid ability		
10. Ability to retrieve and interpret information in clinical data systems		
**Health and society (H&S)**		
1. Responsibility to protect and advance the health and well-being of individuals and populations		
2. Understand factors that contribute to health, illness, disease and success of treatment of populations		
3. Understand the quality assurance system and safety management system of health care in Chinese hospitals		
4. Ability to attach importance to patients’ safety and identify risk factors that are detrimental to patients		
5. Understand the structures and functions of the national health care system in China		
6. Understand global health/ health conditions and issues		
**Professionalism (PF)**		
1. Be familiar with the *Ethic Principles of China Physicians* and related laws and regulations regarding medical industry		
2. Attention to providing humanitarian care for patients		
3. Ability to apply medical ethics in clinical services		
4. Ability to communicated efficiently with patients and their family members		
5. Ability to cooperate and learn from each other		
6. Ability to empathize with the feelings of colleagues, patients and the family members		
7. Understand the factors affecting physicians’ health and wellbeing		
8. Awareness of respect for others		
9. Ability of self-awareness and reflection		
10. Awareness of lifelong learning		
